# Bilateral Neck Dissection Effectively Improves Prognosis of Patients With T3N0M0 Glottic Carcinoma

**DOI:** 10.1002/cam4.71593

**Published:** 2026-02-01

**Authors:** Bixue Huang, Yun Li, Ruihua Fang, Kexing Lv, Zhangfeng Wang, Xiaolin Zhu, Lin Chen, Wenbin Lei

**Affiliations:** ^1^ Department of Otorhinolaryngology Head and Neck Surgery, The First Affiliated Hospital Sun Yat‐sen University Guangzhou China; ^2^ Institute of Otorhinolaryngology Head and Neck Surgery Sun Yat‐sen University Guangzhou China

**Keywords:** glottic carcinoma, lymph node dissection, radiotherapy, SEER database, systemic therapy

## Abstract

**Background:**

To evaluate the effect of bilateral elective node dissection on the prognosis of patients with T3N0M0 glottic carcinoma.

**Methods:**

This retrospective cohort study enrolled two cohorts: patients screened from the Surveillance, Epidemiology, and End Results (SEER) database of the National Cancer Institute, and those treated at the First Affiliated Hospital of Sun Yat‐sen University. Patients screened from the SEER database were divided into untreated, radiotherapy (RT), surgery, and concurrent systemic therapy (ST)/RT groups. Patients from our center were divided into unilateral and bilateral groups based on lymph node dissection. Propensity score‐matching (PSM) was applied to eliminate baseline variations. Kaplan–Meier analysis was used to assess different treatment method effects.

**Results:**

This study retrieved 2027 and 133 patients from the SEER database and our center, respectively, from 2014 to 2022. After PSM, overall survival (OS) and cancer‐specific survival (CSS) improved in the ST/RT (both *p* < 0.001) and surgery (both *p* < 0.001) groups versus the RT group, with no differences between groups (OS, *p* = 0.45; CSS, *p* = 0.84). Patients who underwent elective node dissection (END) had better OS (*p* = 0.025) and CSS (*p* < 0.001) than those without END. No significant difference was observed in OS (*p* = 0.110) between the END and ST/RT groups; however, the END group showed significant improvement in CSS (*p* = 0.007). Patients who underwent bilateral neck dissection had better progression‐free survival than the unilateral group after PSM (*p* = 0.024).

**Conclusion:**

Surgery combined with bilateral node dissection can bring better survival prognosis for patients with T3N0M0 glottic carcinoma.

## 1 Introduction

Laryngeal cancer, the second most common tumor of the respiratory system, has a high incidence and mortality rate worldwide, with an estimated 12,380 new cases and 3820 deaths occurring in 2023 in the United States [1]. Among them, approximately 60% of newly diagnosed laryngeal cancers originate in the glottis. Laryngeal cancer negatively impacts people's health worldwide [2]. In China, the long‐term economic burden caused by laryngeal cancer has attracted attention, due to the rapid development of industrialization and urbanization, coupled with the impact of an aging population, air pollution, and high smoking rate.

Treatment of glottic cancer remains challenging. The selected treatment strategy is vital for prognosis, particularly in patients with advanced glottic carcinoma. The NCCN Clinical Practice Guidelines in Oncology (NCCN guidelines) for head and neck cancers [3] currently recommend several treatment options for patients with T3N0M0 glottic carcinoma. The most commonly selected treatment options in clinical practice include surgery, concurrent systemic therapy (ST) and radiotherapy (RT), and RT alone. In addition, the guidelines recommend induction chemotherapy based on one clinical trial [4]. Although concurrent ST/RT is believed to have comparable survival outcomes with surgery in recent years, surgery remains the preferred treatment for most clinicians.

As the risk of lymph node metastasis in glottic carcinoma is lower than that in supraglottic and subglottic laryngeal carcinoma, the method opted for node dissection among cN0 patients remains ambiguous. The revised NCCN guidelines recommendations for pretracheal and ipsilateral paratracheal lymph‐node dissection in patients with stage N0, allowing for either ipsilateral or bilateral neck dissection. Indeed, one recent retrospective study demonstrated that elective node dissection (END) combined with surgery could effectively improve overall survival (OS) and cancer‐specific survival (CSS) among patients who have not been clinically evaluated for lymph node metastasis [5]. However, there is still a lack of research to indicate which type of lymph node dissection can ensure a better prognosis in T3N0M0 patients; as such, this topic requires further investigation.

Therefore, to provide guidance in this scenario, a retrospective study was conducted, combining data from the Surveillance, Epidemiology, and End Results (SEER) database and our center to explore which treatment method would be more beneficial for the prognosis of patients with T3N0M0 glottic carcinoma to help clinicians develop the best treatment plans for newly diagnosed patients.

## 2 Methods

### 2.1 Data Source

Data for this retrospective cohort study were obtained from the SEER database of the National Cancer Institute and our center. The study protocol was designed in accordance with the Strengthening the Reporting of Observational Studies in Epidemiology statement [6]. The first part of the data was obtained from the SEER database (https://seer.cancer.gov/), which is freely and publicly accessible. Approval to use the data for research was obtained prior to initiating this study. First, the following SEER data were pooled and extracted: year of diagnosis, age, race, sex, grade, stage, laterality, examined lymph nodes (ELNs), time of diagnosis, and survival, using SEER*Stat Software, Version 8.3.5 (https://seer.cancer.gov/). Second, clinical data from patients who visited the Department of Otorhinolaryngology at the First Affiliated Hospital of Sun Yat‐sen University were collected (hereafter referred to as the SYSU database).

### 2.2 Ethical Considerations

This study was performed in line with the principles of the Declaration of Helsinki and was approved by the Ethics Committee for Research and Publication of Sun Yat‐sen University. Patients' data were kept anonymous following extraction from the original medical records. As all data were anonymized and collected retrospectively, informed consent was not required from the subjects.

### 2.3 Patient Selection

In total, 23,535 patients diagnosed with glottic cancer between 2004 and 2020 from the SEER database and 982 patients between 2015 and 2023 from the SYSU database were screened. Because SEER does not consistently distinguish clinical and pathological TNM staging across most years, patients were selected according to the AJCC‐defined T3N0M0 stage recorded in the SEER database, which represents the standardized staging information provided by SEER. Only patients with complete clinical and survival data were included. Patients selected from the SEER database were divided into the RT, surgery, and ST/RT groups, according to their treatment regimens. The surgery group was further divided into two groups based on whether neck dissection was performed. To address the potential concern that the SEER N0 category may include both clinical and pathological N0 cases, we performed an additional validation analysis using the only 2 years in which SEER explicitly recorded clinical staging (cT3cN0cM0, 2016–2017). These cases were re‐selected and reanalyzed independently.

**FIGURE 1:**
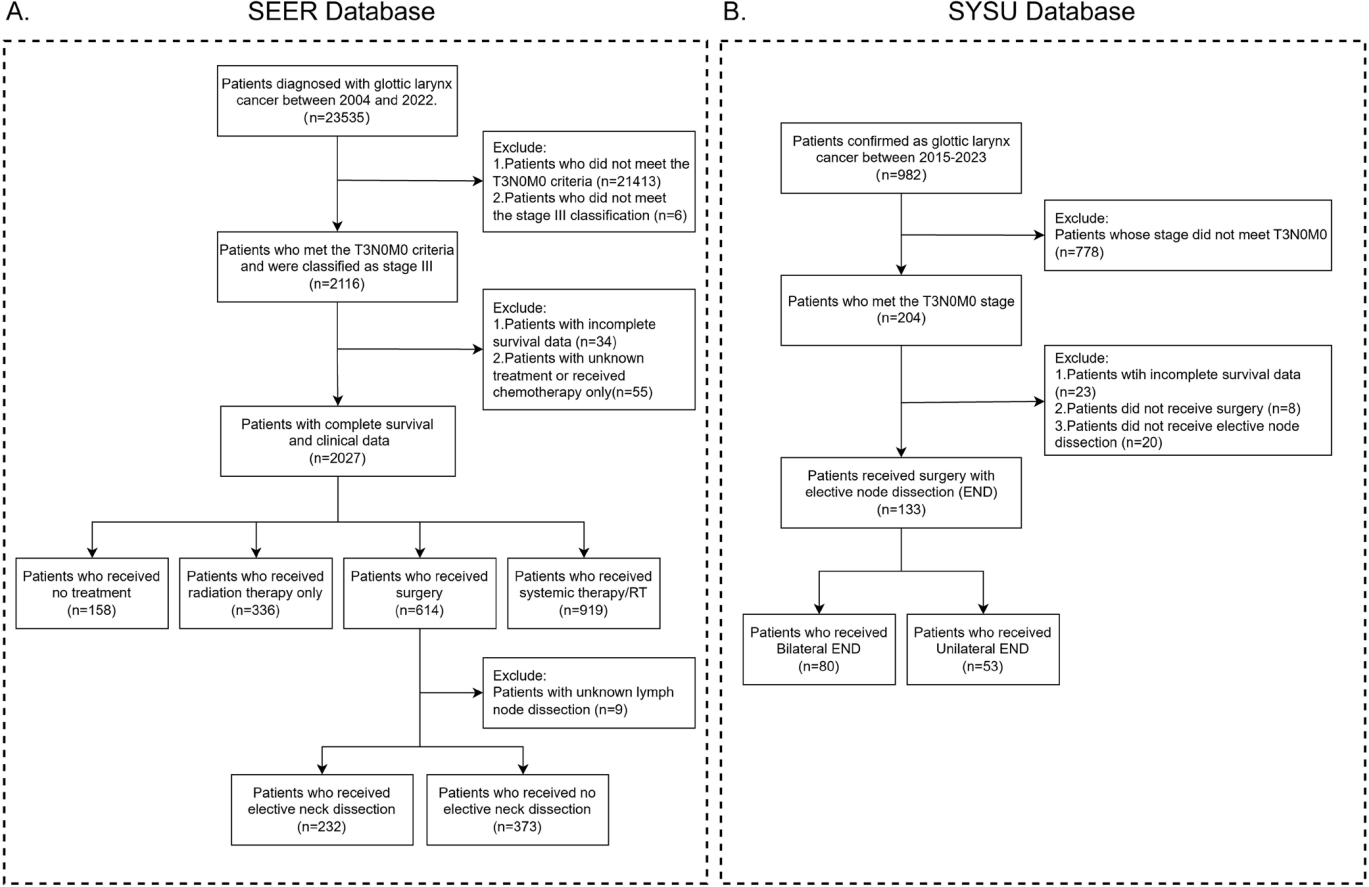
Flowchart of data selection. Patient screening criteria in the SEER database (A) and in SYSU database (B).

Because the SEER database does not provide detailed information regarding the specific method of lymph node dissection, surgical records from the SYSU clinical center were collected to further examine the impact of different lymph node dissection strategies on patient prognosis. For the SYSU database, after initial screening, patients who underwent surgery with concurrent neck dissection were further selected and divided into the unilateral and bilateral groups based on the type of dissection. The detailed screening procedure is illustrated in Figure 1.

### 2.4 Selection of Lymph Node Dissection, Complication Management, and Follow‐Up

For the patients included in our clinical center, the choice of surgical methods was determined by the clinician based on the size and location of the tumor and the patient's personal health condition. The clinicians who performed the surgery were all senior surgeons with more than 10 years of experience in laryngeal cancer surgery. The extent of dissection on each side included levels II–IV, and central lymph node dissection was routinely performed at the same time. Specimens were taken for intraoperative frozen pathological examination each time. Surgery was continued after ensuring negative margins. The patients with pharyngocutaneous fistula after operation were treated with transnasal negative pressure therapy [7]. All surgical complications were treated at the same time after operation. Patients were followed up at the hospital outpatient clinic at 1, 3, 6, 9 and 12 months after surgery. Follow‐up was then performed annually after 1 year and every 3 months in between visits by telephone.

### 2.5 Statistical Analysis

The major endpoints for data from the SEER and SYSU databases were OS and progression‐free survival (PFS), respectively, while the secondary endpoint was CSS. OS, CSS, and PFS events were defined as death; death due to glottic cancer; and death, recurrence, or metastasis, respectively.

Patients from the SEER database were divided into four groups: untreated, RT, surgery, and ST/RT. Descriptive parameters among groups were compared using the chi‐square or Fisher's exact tests. Subsequently, according to different comparison needs, we adopted propensity‐score matching (PSM) processing for different comparison groups. PSM was applied to minimize selection bias among baseline variables between the groups, including patient age, sex, race, laterality, and grade. In the matching of patients in the END versus non‐END groups, whether the patient received RT was additionally included. All matching processes were performed using the R package “pm3.” The “optimal” method and 1:1 matching ratio were selected in the PSM. PSM is a commonly used tool that can greatly minimize the selection bias of unequally sized treatment groups, while preserving an adequate sample size [8, 9]. Chi‐square tests and Fisher's exact tests were performed on the baseline variables after PSM. Further, Kaplan–Meier analysis was used to compare OS and CSS among groups, using the log‐rank test before and after PSM. Multivariate Cox regression hazard regression analyses were performed for patient age, sex, race, laterality, grade, and disease stage to calculate the hazard ratio (HR) and determine independent prognostic factors. According to the contribution degree of each influencing factor to the outcome variable in the model, each value level of each influencing factor was assigned to construct the nomogram. The nomogram and forest map were subsequently used for evaluation. In the surgery group, the X‐tile program (http://www.tissuearray.org) was applied to determine the optimal cutoff point for the number of ELNs providing the greatest benefit regarding prognosis, with minimum *p* values [10].

In the analysis of data from the SYSU database, PSM was also applied to analyze the descriptive parameters of patients with unilateral and bilateral neck dissection. In addition to the variables used in the SEER database analysis, ECOG Performance Status was also included. Multivariable Cox proportional hazards regression analysis was also performed. Covariates included in the adjusted model were selected based on clinical relevance, including gender, age, surgical method, grade, laterality, ECOG performance status, and receipt of adjuvant radiotherapy. The data before and after matching were analyzed using chi‐square or Fisher's exact tests and Kaplan–Meier analysis to compare PFS between two groups. The cutoff values were calculated using X‐lite, as detailed previously.

All analyses were performed using SPSS software (version 26.0) and R software (version 4.3.1); *p* values < 0.05 were considered statistically significant.

## 3 Results

### 3.1 Baseline Characteristics and PSM

**FIGURE 2:**
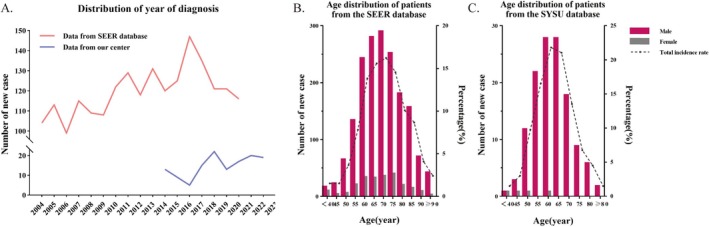
(A) The distribution of the year of onset of the patients. The age of onset of patients with different genders (B) in SEER database and (C) in SYSU database.

Based on the inclusion and exclusion criteria, 2027 and 133 patients with T3N0M0 glottic laryngeal cancer were screened from the SEER and SYSU databases, respectively (Figure 1). The analysis of the diagnosis year revealed an overall upward trend in the disease incidence from the 2000s to the 2010s, with a slight decline over the last 5 years. Regarding onset age, patients extracted from the SEER database were primarily concentrated at approximately 70 years old, whereas patients from our center were relatively young, at approximately 60 years old. However, both datasets suggested a higher disease prevalence in men (Figure 2).

First, we analyzed data from the SEER database. Patients were divided into untreated, RT, surgery, and concurrent ST/RT groups based on the treatment method. The baseline clinical features are shown in Table 1. The ST/RT group had the most patients, followed by the surgery group (158, untreated; 336, RT; 614, surgical; and 919, ST/RT). The years since diagnosis were more recent in the ST/RT group versus the other three groups, indicating that, compared to other treatment methods, the clinical treatment strategy for such patients has gradually become to include concurrent ST/RT at a greater proportion in recent years. The other significant differences among the four groups were mainly found in age, grade, and stage (*p* < 0.001). Regarding age, patients aged ≥ 65 years accounted for the majority of patients in the untreated (74.05%) and RT groups (71.43%); however, the proportions in the surgery (53.75%) and ST/RT groups (48.75%) were similar to those in the younger groups. Regarding grade, despite significant differences in the composition of the four groups, all groups showed moderate differentiation. Additionally, most patients were male (87.47%), Caucasian (76.32%), and not a pair laterality (75.28%).

**TABLE 1:** Clinical characteristics before and after propensity score matching (PSM), data from SEER database.

Variables	Before PSM						After PSM					
	Total (*n* = 1869)	Method			Statistic	*p*	Total (*n* = 1008)	Method			Statistic	*p*
		Radiation therapy (*n* = 336)	Surgery (*n* = 614)	Systemic therapy/RT (*n* = 919)				Radiation therapy (*n* = 336)	Surgery (*n* = 336)	Systemic therapy/RT (*n* = 336)		
Sex, *n* (%)					*χ* ^2^ = 1.40	0.578					*χ* ^2^ = 1.45	0.485
Male	1630 (87.21)	297 (88.39)	529 (86.16)	804 (87.49)			889 (88.19)	297 (88.39)	291 (86.61)	301 (89.58)		
Female	239 (12.79)	39 (11.61)	85 (13.84)	115 (12.51)			119 (11.81)	39 (11.61)	45 (13.39)	35 (10.42)		
Age, *n* (%)					*χ* ^2^ = 51.22	< 0.001					*χ* ^2^ = 0.03	0.986
< 65	851 (45.53)	96 (28.57)	284 (46.25)	471 (51.25)			291 (28.87)	96 (28.57)	98 (29.17)	97 (28.87)		
≥ 65	1018 (54.47)	240 (71.43)	330 (53.75)	448 (48.75)			717 (71.13)	240 (71.43)	238 (70.83)	239 (71.13)		
Race, *n* (%)					*χ* ^2^ = 6.42	0.170					*χ* ^2^ = 0.58	0.966
White	1424 (76.19)	263 (78.27)	479 (78.01)	682 (74.21)			797 (79.07)	263 (78.27)	270 (80.36)	264 (78.57)		
Black	341 (18.25)	55 (16.37)	110 (17.92)	176 (19.15)			157 (15.58)	55 (16.37)	49 (14.58)	53 (15.77)		
Others	104 (5.56)	18 (5.36)	25 (4.07)	61 (6.64)			54 (5.36)	18 (5.36)	17 (5.06)	19 (5.65)		
Laterality, *n* (%)					*χ* ^2^ = 7.32	0.292					—	0.672
Left	220 (11.77)	39 (11.61)	63 (10.26)	118 (12.84)			110 (10.91)	39 (11.61)	37 (11.01)	34 (10.12)		
Right	224 (11.99)	43 (12.80)	67 (10.91)	114 (12.40)			119 (11.81)	43 (12.80)	32 (9.52)	44 (13.10)		
Bilateral	17 (0.91)	2 (0.60)	3 (0.49)	12 (1.31)			6 (0.60)	2 (0.60)	1 (0.30)	3 (0.89)		
Not a paired site	1408 (75.33)	252 (75.00)	481 (78.34)	675 (73.45)			773 (76.69)	252 (75.00)	266 (79.17)	255 (75.89)		
Grade, *n* (%)					*χ* ^2^ = 32.99	< 0.001					*χ* ^2^ = 1.60	0.991
Well differentiated	244 (13.06)	44 (13.10)	91 (14.82)	109 (11.86)			130 (12.90)	44 (13.10)	44 (13.10)	42 (12.50)		
Moderately differentiated	837 (44.78)	150 (44.64)	297 (48.37)	390 (42.44)			451 (44.74)	150 (44.64)	154 (45.83)	147 (43.75)		
Poorly undifferentiated	206 (11.02)	31 (9.23)	85 (13.84)	90 (9.79)			95 (9.42)	31 (9.23)	31 (9.23)	33 (9.82)		
Undifferentiated	5 (0.27)	1 (0.30)	2 (0.33)	2 (0.22)			2 (0.20)	1 (0.30)	1 (0.30)	0 (0.00)		
Unknown	577 (30.87)	110 (32.74)	139 (22.64)	328 (35.69)			330 (32.74)	110 (32.74)	106 (31.55)	114 (33.93)		

Abbreviation: *χ* ^2^, chi‐square test.

Based on the subsequent analysis requirements, we performed PSM separately on the three treatment groups in the SEER database (Table 1), on whether END was performed in the surgical group and ST/RT group (Table S2), and on the two surgical groups (Table S3). After PSM, no significant differences in baseline variables were observed among groups (*p* > 0.05), indicating that the variables were well balanced after matching.

According to data from the SYSU database, patients were divided into bilateral (80/133) and unilateral (53/133) node dissection groups based on the node dissection method. Most patients were male (96.99%), aged < 65 years old (74.48%), had bilateral laterality (75.19%), moderate differentiation (60.15%), and did not receive adjuvant RT (74.44%). However, differences in the surgical methods used were observed between the groups (*p* = 0.002). Partial laryngectomy was performed in 65% of patients with bilateral neck dissection, and 86.79% of patients in the unilateral group. In addition, the physical condition of patients at admission was assessed, and the ECOG Performance Status score showed no significant differences between the groups. The majority of patients were able to perform normal activities and were independent in daily living at the time of admission. Similarly, PSM was performed to eliminate differences between groups (Table 2).

**TABLE 2:** Clinical characteristics before and after propensity score matching (PSM), data from SYSU database.

Variables	Before PSM					After PSM				
	Total (*n* = 133)	Elective node dissection		Statistic	*p*	Total (*n* = 104)	Elective node dissection		Statistic	*p*
		Bilateral (*n* = 80)	Unilateral (*n* = 53)				Bilateral (*n* = 52)	Unilateral (*n* = 52)		
Year of diagnosis, *M* (*Q*_1_, *Q*_3_)	2019.00 (2017.00, 2021.00)	2019.50 (2017.00, 2021.00)	2018.00 (2017.00, 2021.00)	*Z* = −1.015	0.310	2018.00 (2017.00, 2020.25)	2018.00 (2017.00, 2020.00)	2018.00 (2017.00, 2021.00)	*Z* = −0.387	0.699
Sex, *n* (%)				*χ* ^2^ = 0.88	0.347				*χ* ^2^ = 1.37	0.241
Male	129 (96.99)	79 (98.75)	50 (94.34)			101 (97.12)	52 (100.00)	49 (94.23)		
Female	4 (3.01)	1 (1.25)	3 (5.66)			3 (2.88)	0 (0.00)	3 (5.77)		
Age, *n* (%)				*χ* ^2^ = 0.15	0.703				*χ* ^2^ = 2.74	0.098
< 65	97 (73.48)	59 (74.68)	38 (71.70)			81 (77.88)	44 (84.62)	37 (71.15)		
≥ 65	35 (26.52)	20 (25.32)	15 (28.30)			23 (22.12)	8 (15.38)	15 (28.85)		
Laterality, *n* (%)				*χ* ^2^ = 0.13	0.939				*χ* ^2^ = 0.20	0.907
Left	19 (14.29)	12 (15.00)	7 (13.21)			15 (14.42)	8 (15.38)	7 (13.46)		
Right	14 (10.53)	8 (10.00)	6 (11.32)			12 (11.65)	7 (13.46)	6 (11.54)		
Bilateral	100 (75.19)	60 (75.00)	40 (75.47)			76 (73.08)	37 (71.15)	39 (75.00)		
Grade, *n* (%)				*χ* ^2^ = 3.496	0.174				*χ* ^2^ = 1.49	0.474
Well differentiated	28 (21.05)	20 (25.00)	8 (15.09)			19 (18.45)	11 (21.15)	8 (15.38)		
Moderately differentiated	80 (60.15)	43 (53.75)	37 (69.81)			65 (63.11)	30 (57.69)	36 (69.23)		
Poorly differentiated	25 (18.8)	17 (21.25)	8 (15.09)			19 (18.45)	11 (21.15)	8 (15.38)		
Surgical method, *n* (%)				—	0.002				*χ* ^2^ = 0.00	1.000
Local tumor excision	1 (0.75)	0 (0.00)	1 (1.89)			0 (0.00)	0 (0.00)	0 (0.00)		
Partial laryngectomy	98 (73.68)	52 (65.00)	46 (86.79)			92 (88.46)	46 (88.46)	46 (88.46)		
Total laryngectomy	34 (25.56)	28 (35.00)	6 (11.32)			12 (11.54)	6 (11.54)	6 (11.54)		
Adjuvant radiotherapy, *n* (%)				*χ* ^2^ = 1.07	0.301				*χ* ^2^ = 0.23	0.631
Yes	34 (25.56)	23 (28.75)	11 (20.75)			22 (21.15)	12 (23.08)	10 (19.23)		
No	99 (74.44)	57 (28.75)	42 (79.25)			82 (78.85)	40 (76.92)	42 (19.23)		
ECOG performance status, *n* (%)				—	0.951				—	0.579
0	72 (54.14)	42 (52.50)	30 (56.60)			53 (50.96)	23 (44.23)	30 (57.69)		
1	54 (40.60)	34 (42.50)	20 (37.74)			44 (42.31)	25 (48.08)	19 (36.54)		
2	5 (3.76)	3 (3.75)	2 (3.77)			5 (4.81)	3 (5.77)	2 (3.85)		
3	2 (1.50)	1 (1.25)	1 (1.89)			2 (1.92)	1 (1.92)	1 (1.92)		

*Note:* “—” denotes Fisher exact.

Abbreviation: *χ* ^2^, chi‐square test.

### 3.2 Surgical Treatment Improves the Survival of Patients

**FIGURE 3:**
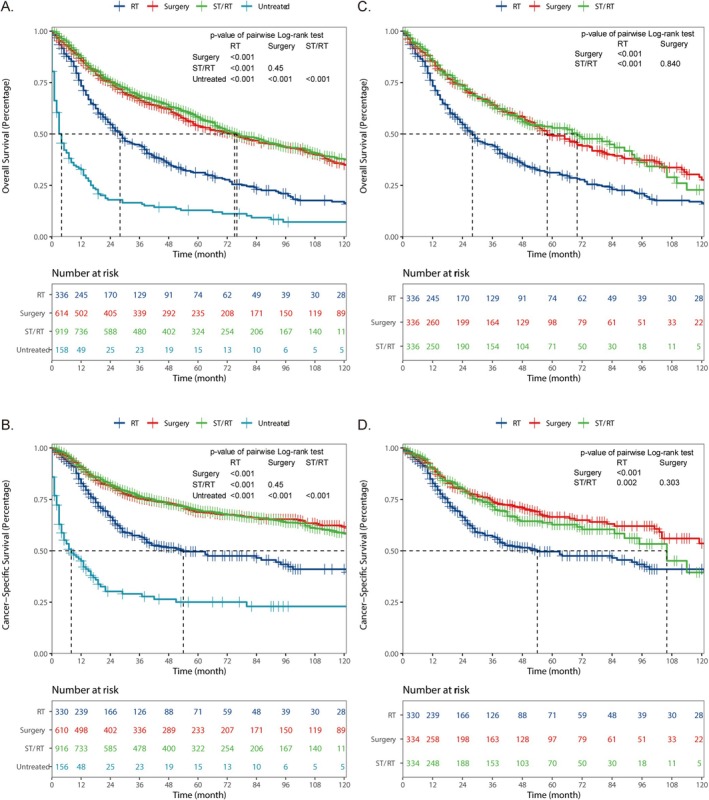
Kaplan–Meier survival curves for OS (A, C) and CSS (B, D) among different treatment groups in raw data (A, B), data after PSM (C, D).

We first analyzed the OS and CSS survival data of patients under different treatment regimens from the SEER database. The results revealed that surgery could significantly improve the OS (*p* < 0.001) and CSS (*p* < 0.001) compared with the untreated group (Figure 3). Further, the other two treatments also improved the prognosis of patients to varying degrees. Surgery was associated with significantly superior outcomes compared to the RT group (OS, *p* < 0.001; CSS, *p* < 0.001) and has a comparable effect to the ST/RT group (OS, *p* = 0.45; CSS, *p* = 0.45). The same conclusion could be drawn with the data after PSM. To further minimize potential bias related to clinical versus pathological staging, we performed an additional analysis restricted to patients from years in which explicit clinical TNM information was available and extracted those recorded as cT3cN0cM0. This clinically staged subgroup demonstrated survival patterns consistent with the overall cohort, supporting the robustness of the primary findings (Figure S1; Tables S4 and S5).

**FIGURE 4:**
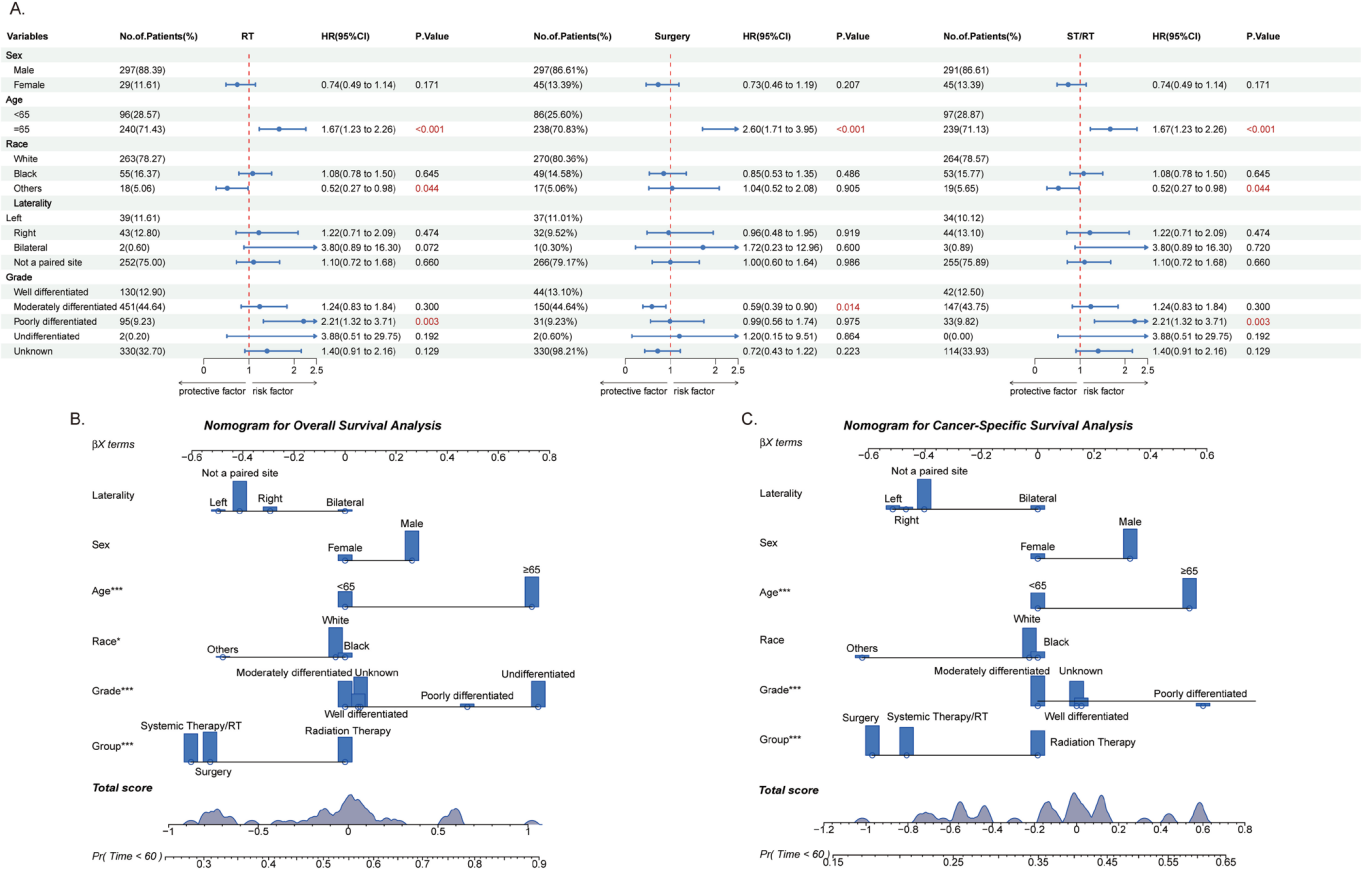
Forest plot for the overall survival subgroup analysis of the prognostic value of RT, Surgery and ST/RT group after PSM (A). Construction of OS (B) and CSS (C) nomogram.

In addition, univariate Cox regression analyses of the three treatment groups showed similarities and differences in the various influencing factors among the groups (Figure 4). Age ≥ 65 years was a risk factor shared by all three groups (*p* < 0.001). In both the RT and ST/RT group, poor differentiation (*p* = 0.003) was identified as a risk factor, while moderate differentiation (*p* = 0.014) was a risk factor in the surgery group. In contrast, other race (*p* = 0.044) appeared to be a more important risk factor in the ST/RT group. We subsequently established an OS and CSS nomogram model for 5‐year survival (Figure 4).

### 3.3 Elective Neck Dissection Further Improves the Survival Prognosis

**FIGURE 5:**
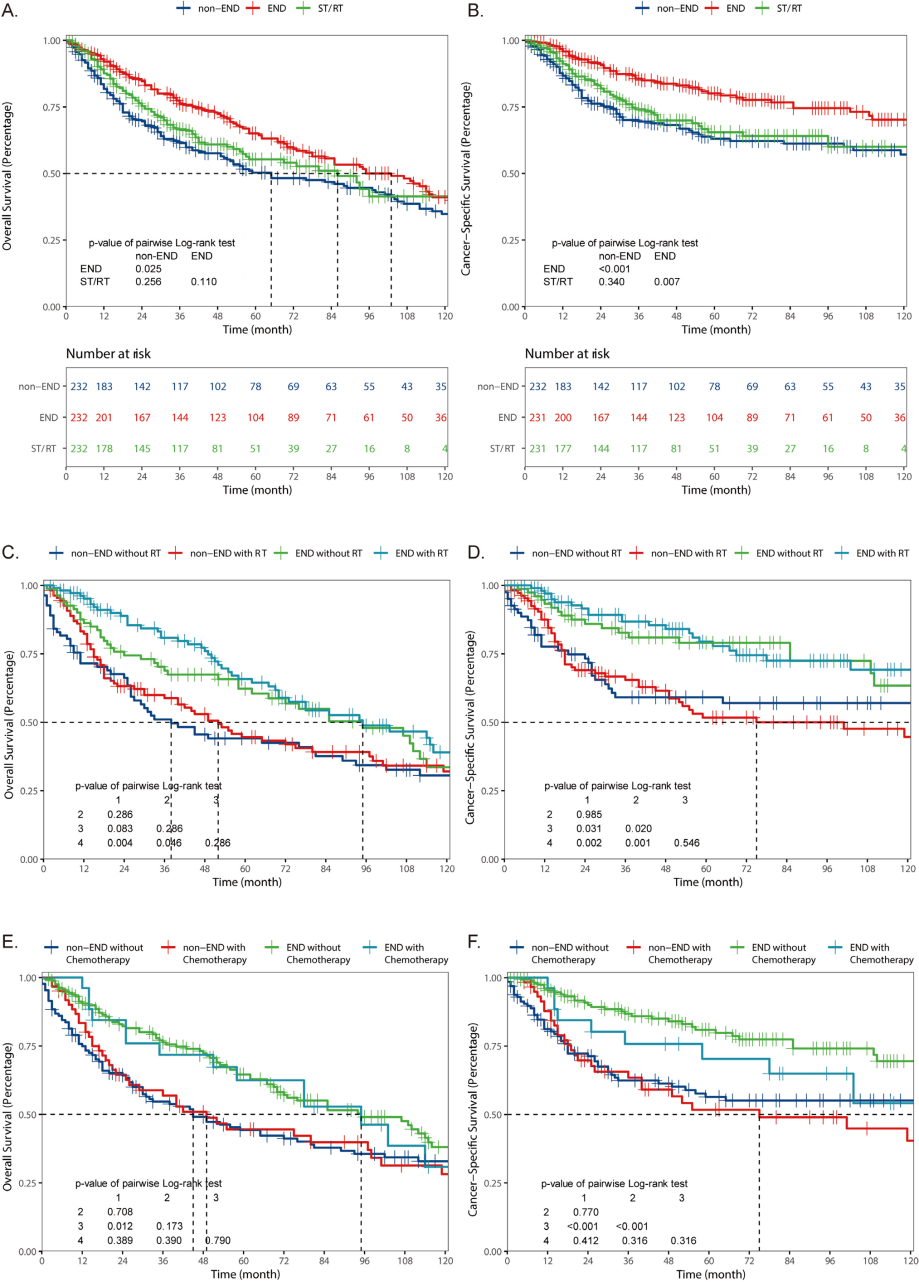
Kaplan–Meier survival curves for OS (A, C, E) and CSS (B, D, F) among END subgroup and ST/RT after PSM (A, B), and among subgroups with different END and RT methods (C, D) and different END and chemotherapy methods (D, F). (In graphs C and D, 1 represents non‐END without RT, 2 represents non‐END with RT, 3 represents END without RT, and 4 represents END with RT. In graphs E and F, 1 represents non‐END without Chemotherapy, 2 represents non‐END with Chemotherapy, 3 represents END without Chemotherapy, and 4 represents END with Chemotherapy.)

Patients in the surgery group were further divided into the non‐END group and the END group for comparison with the ST/RT group. After performing PSM on the three groups of data, survival analysis comparison was conducted (Figure 5, Table S3). In the surgery group, patients who underwent END had significantly better OS (*p* = 0.025) and CSS (*p* < 0.001) than those who did not undergo END. Although the ST/RT group can extend the median survival time of OS compared to the non‐END group (non‐END: 65.00[50.00–102.00]; ST/RT: 103.00 [78.00–122.00]), there is no statistically significant difference in OS and CSS comparisons (OS: *p* = 0.256; CSS: *p* = 0.340). Further, a comparison between the END and ST/RT groups revealed no significant differences in OS (*p* = 0.110); however, the END group showed a significant improvement in CSS (*p* = 0.007).

Next, we perform another PSM on the data from the two surgery groups. The results indicated that END significantly improved the prognosis of patients in terms of OS (*p* = 0.0016) and CSS (*p* < 0.001) (Figure S2). Further analysis was performed to assess the impact of additional RT and CT in the surgery group (Figure 5). Our finding that additional RT and CT did not significantly improve OS and CSS in patients in either the non‐END or END groups. In the non‐END group, additional RT or CT is beneficial, as it can improve the median survival time of patients. However, this benefit is much smaller compared to the survival advantage provided by undergoing END.

**FIGURE 6:**
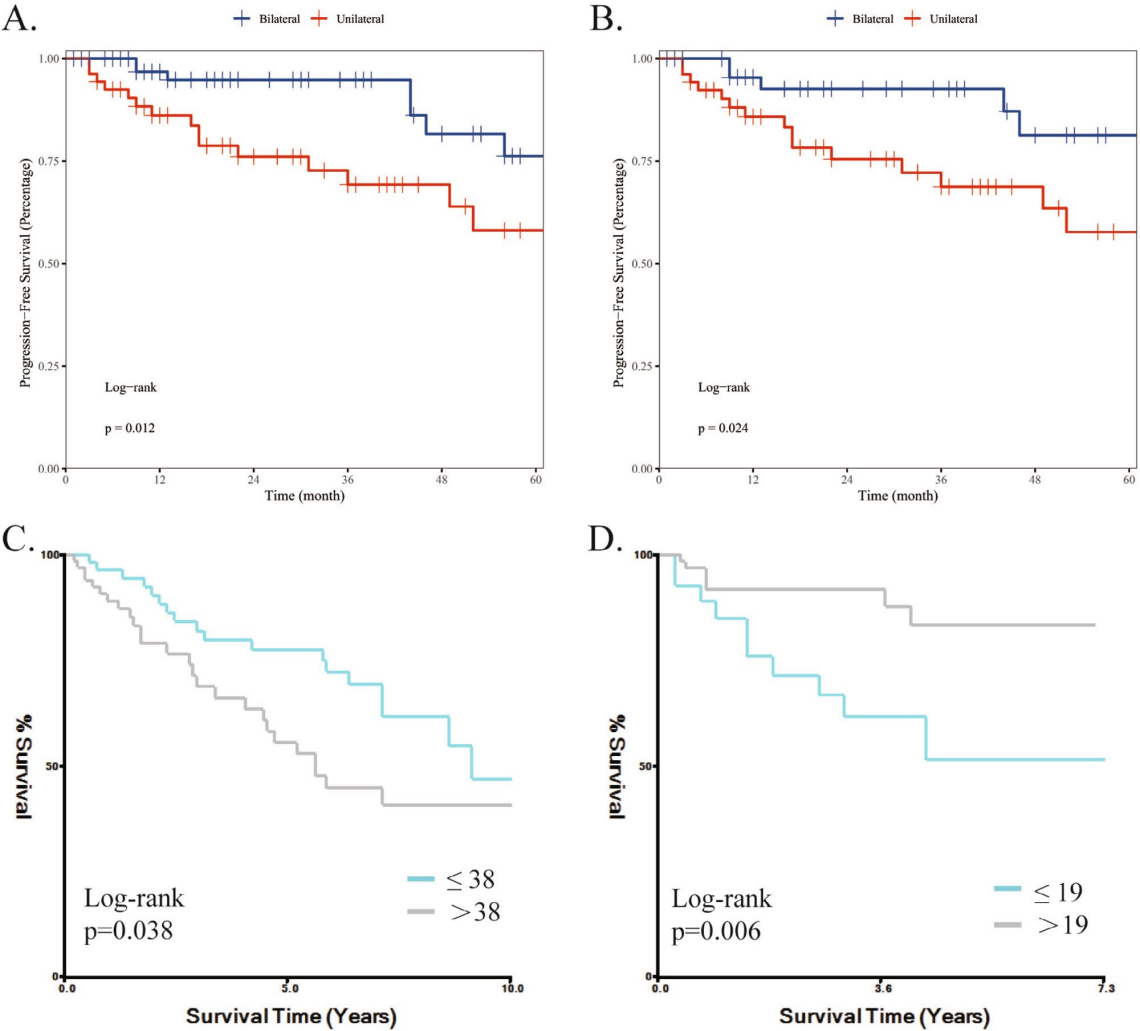
Kaplan–Meier survival curves for PFS between bilateral END group and unilateral group in raw data (A), and data after PSM (B). The optimal examined lymph nodes cut‐off values according to overall survival by X‐tile analysis for data from SEER database (C), and SYSU database (D).

To further evaluate the impact of different neck dissection strategy extent on survival outcomes, we analyzed data from the SYSU database whose patients were undergoing unilateral or bilateral neck dissection. Multivariable Cox regression analysis adjusted for clinicopathological covariates demonstrated a significant association between neck dissection strategy and prognosis (*p* < 0.05) (Table S6). Subsequent Kaplan–Meier survival analysis revealed that patients receiving bilateral neck dissection had significantly improved PFS compared to those with unilateral dissection (*p* = 0.012) (Figure 6). This survival advantage remained consistent after PSM (*p* = 0.024), reinforcing the prognostic value of bilateral neck dissection.

Data on the number of regional nodes examined for all patients who underwent surgery with neck dissection from the two databases was pooled, and a cutoff analysis was performed (Figure 6). The results from the SEER database suggested that the cutoff value was 38; the Kaplan–Meier curve suggested that when the number of lymph nodes dissected exceeded 38, the OS of patients decreased significantly (*p* = 0.038). Data from the SYSU database suggested that OS was significantly lower, with fewer than 19 retrieved lymph nodes (*p* = 0.006).

Information on adverse effects among the different groups was also collected (Table 3). Adverse reactions in the bilateral END group occurred throughout the body; however, adverse reactions in the unilateral dissection group were primarily confined to the surgical site and respiratory system.

**TABLE 3:** Adverse effects in different END subgroups.

Adverse event	Elective node dissection	
	Bilateral (%)	Unilateral (%)
Respiratory tract infection		1 (0.7)
Pneumonia	5 (3.7)	3 (2.2)
Atelectasis	1 (0.7)	2 (1.5)
Pleural effusion		1 (0.7)
Emphysema	1 (0.7)	
Pulmonary embolism	3 (2.2)	
Acute heart failure	1 (0.7)	
Dyspnea		1 (0.7)
Laryngeal edema		1 (0.7)
Pharyngocutaneous fistula	2 (1.5)	3 (2.2)
Hematoma	1 (0.7)	1 (0.7)
Hemorrhage	1 (0.7)	3 (2.2)
Delayed Wound healing	1 (0.7)	1 (0.7)
Wound infection	3 (2.2)	1 (0.7)
Pneumoderm		3 (2.2)
Chylous leakage	1 (0.7)	
Mandibular nerve injury	1 (0.7)	
Liver function impairment	2 (1.5)	2 (1.5)
Impairment of kidney	1 (0.7)	
Uarhritis	2 (1.5)	
Hypokalemia	2 (1.5)	
Thrombocytopenia	1 (0.7)	
Lumbago	1 (0.7)	
Enterogastritis	1 (0.7)	
Allergic dermatitis	2 (1.5)	

## 4 Discussion

The NCCN guidelines provide several concurrent treatment options, including concurrent ST/RT, RT, and surgery, which are commonly used in clinics, and induction chemotherapy and clinical trials for T3N0M0 glottic carcinoma treatment [4, 11, 12]. In the present study, analysis of data from the SEER database indicated that surgery and ST/RT significantly improved patient survival. By integrating real‐world data from our center, we further demonstrated that surgical treatment combined with neck dissection—particularly bilateral dissection—was associated with more favorable survival outcomes, with bilateral lymph node dissection showing a clear advantage in PFS.

Many previous studies have shown that concurrent CT and RT could achieve the same prognosis as surgery in patients with advanced glottic carcinoma [12, 13, 14, 15], and could be considered an alternative treatment to surgery, as confirmed in our study, which revealed no significant difference in OS or CSS between groups. Nevertheless, although the long‐term oncological outcomes of ST/RT are comparable to those of surgery, systemic therapy carries substantial treatment‐related toxicity. For example, systemic symptoms of ST are more severe, and patients may have worsened quality of life; moreover, this approach is commonly associated with acute and delayed toxicity [16]. The side effects include anemia [17], neutropenia [18], nausea, vomiting [19], diarrhea, oral mucosal inflammation [20], polyneuropathy, and specific toxicities such as ototoxicity [21], nephrotoxicity [18], pulmonary toxicity, and neurotoxicity. The dysphagia and weight loss risks also substantially increase with age [18], commonly leading to fatigue, functional limitations, and decreased quality of life. Conversely, surgical treatment carries fewer complications. With improvements in surgical methods and technological progress, adverse reactions to surgical treatment are primarily confined to the surgical area. Furthermore, patients' quality of life typically remains stable following the perioperative period.

Many studies of malignant tumors have recognized that adequate lymph node dissection can improve survival prognosis. In an analysis of 4224 patients with esophageal cancer in the SEER database, an ELNs of 18 or more was associated with a survival benefit [22]. Even without lymph node metastasis, a certain number of elective lymph node dissections is a protective factor for the prognosis of patients. The NCCN guidelines consider dissection of fewer than 12 lymph nodes to be a high‐risk factor in patients with T3N0M0 colon cancer [23]. Some results have been shown in early‐stage cervical cancer [24] and fallopian tube cancer [25], with favorable PFS and OS associated with more than a certain number of lymph nodes retrieved. Similar studies have been conducted in laryngeal cancer. For patients with early T1‐2N0 supraglottic carcinoma, ELNs > 10 can significantly improve the OS [26]. In line with these findings, our analysis revealed that ELNs > 19 was associated with significantly improved survival in T3N0M0 glottic carcinoma. Interestingly, from the SEER database, we found that excess lymph node dissection (ELN ≥ 38) also had a negative impact on survival outcomes.

In the SEER database, most T3N0M0 cases were coded as “nodes not examined” or “unknown,” meaning that the cohort essentially reflects clinically node‐negative rather than pathologically confirmed pN0 disease. Because SEER does not consistently separate cTNM from pTNM staging, we additionally analyzed the only years in which explicit clinical staging was available (cT3cN0cM0). The survival patterns in this clinically staged subgroup were highly consistent with the overall results, supporting the robustness and clinical relevance of our findings.

After a more detailed grouping of surgery with or without END, we found that END significantly improved CSS compared with the ST/RT group (*p* = 0.006). Indeed, elective lymph node dissection among patients with cancer is recognized by most surgeons, with many studies showing its importance. For example, in patients with negative cervical lymph nodes and T1–2 supraglottic cancer, lymph node dissection was found to be significantly associated with a more favorable prognosis [27]. Patients with human papillomavirus‐positive T1–2 cN0 oropharyngeal cancer could also benefit from END in terms of OS [28]. Moreover, level II END is worthwhile in patients with cT3–4N0 parotid gland cancer [29]. Therefore, we believe that surgery combined with neck dissection is the optimal option. Furthermore, we among the researchers are more concerned about the differences in the sides of the lymph nodes dissected. Cervical lymph node dissection benefits may be associated with the distribution of cervical lymph nodes. Approximately 150–300 of the estimated 800 lymph nodes in the human body are located in the neck region [30, 31], while the cervical lymph nodes are symmetrically distributed along the midline of the body, separate but connected [32]. Even in patients with no lymph node metastasis, there is a 15%–20% risk of latent metastasis [33]. The ipsilateral lymph nodes are generally dissected for unilateral head and neck cancers; however, for T3 glottic carcinoma, there is currently no consensus on whether dissection should be performed on the main side of the lesion or bilaterally.

For further exploration, we collected data from our center for research and analysis on how to select the optimal extent of lymph node dissection for patient benefit. These results confirm that bilateral neck dissection is superior to unilateral neck dissection, as reflected in the superior PFS of these patients. Following bilateral neck dissection, patients had significantly lower odds of in situ recurrence and detection of neck‐lymphatic or distant metastases. Based on the cutoff analysis data and the number of lymph nodes examined in our center, we concluded that the dissection of more than 19 lymph nodes could improve the PFS of patients. However, this conclusion may be due to latent metastasis in the cervical lymph nodes in patients with T3 stage, which is not detected by imaging or pathology [33]. This undetected metastasis may reactivate the disease later, reducing survival time. Therefore, RT and CT, in addition to surgery and node dissection, may improve the median survival time of patients; moreover, these methods are adjuvant treatments for small, undetected metastases [34]. One notable difference in data from the SEER database was that when the number of lymph nodes dissected exceeded 38, the OS of patients decreased. This may be due to individual disease characteristics. For example, the number of dissected lymph nodes may be a prognostic indicator in patients with more reactive lymph nodes. Previous studies have also found that high numbers of ELNs in patients with advanced head and neck squamous cell carcinoma correlate with worsened patient prognosis [35]. The increase in reactive lymph nodes not only increases the probability of metastasis but also of missing serial pathological sections, leading to decreased survival.

One of the main arguments against elective neck dissection is the perception that it is associated with a higher risk of subsequent complications; however, proponents of neck dissection have shown that END can be performed by experienced surgeons without increased morbidity and complications. Therefore, we recommend that if a patient's physical condition permits, surgery with bilateral neck dissection can improve patient benefits.

In the SEER database, most T3N0M0 cases were recorded as having “no nodes examined” or “unknown” lymph node status, indicating that these patients were essentially staged as clinical N0. Therefore, the patients included in our analysis were primarily based on clinical staging rather than pathological confirmation, avoiding potential bias from postoperative pathological upstaging. However, because SEER does not consistently distinguish between clinical and pathological staging across many years, we additionally extracted a cohort with explicitly recorded clinical staging (cT3cN0cM0). The results in this subset were consistent with the overall findings, supporting the robustness of our conclusions.

Admittedly, there are still some shortcomings in this study. The possibility of subglottic extension in T3 patients in this study, who have an increased risk of paratracheal lymph node metastasis, is also worth exploring. Central lymph node excision is routinely performed in our center, but a detailed description of this region is lacking in the SEER database. Besides, this study was limited to a single center, and the sample size was not sufficiently large. Although data have confirmed that bilateral neck dissection is superior to unilateral neck dissection, the level of clinical evidence from retrospective single‐center studies is still insufficient. In the future, we plan to conduct a multicenter, prospective study to provide higher‐level clinical evidence to assist physicians in making clinical decisions.

## 5 Conclusions

For patients with T3N0M0 glottic carcinoma, surgery combined with END results in a better prognosis and bilateral node dissection is associated with better PFS.

## Author Contributions

**Bixue Huang:** conceptualization (equal), data curation (equal), formal analysis (lead), writing – original draft (lead). **Yun Li:** conceptualization (equal), formal analysis (supporting), writing – review and editing (supporting). **Ruihua Fang:** methodology (equal), software (equal). **Kexing Lv:** methodology (equal). **Zhangfeng Wang:** data curation (equal), supervision (equal). **Xiaolin Zhu:** data curation (equal), supervision (equal). **Lin Chen:** conceptualization (equal), data curation (equal), supervision (equal), writing – review and editing (supporting). **Wenbin Lei:** conceptualization (equal), funding acquisition (lead), supervision (lead), writing – review and editing (lead).

## Funding

This work was supported by the Basic and Applied Research Foundation of Guangdong Province (No. 2022B1515130009), National Natural Science Foundation of China (Nos. 81972528 and 82273053), and 5010 Clinical Research Program of Sun Yat‐sen University (No. 2017004).

## Ethics Statement

This study was approved by the Ethics Committee for Research and Publication of Sun Yat‐sen University. Patients' data were kept anonymous after extraction from the original medical records.

## Consent

As all data were anonymized and collected retrospectively, informed consent was not required from the subjects.

## Conflicts of Interest

The authors declare no conflicts of interest.

## Supporting information


**Figure S1:** Kaplan–Meier survival curves for patient with cT3cN0cM0 in OS (A, C) and CSS (B, D) among different treatment group in raw data (A, B), data after PSM (C, D).

**Figure S2:** Kaplan–Meier survival curves for patient with and without END in OS (A) and CSS (B), data after PSM.

**Table S1:** Clinical characteristics in raw data from SEER database.
**Table S2:** Clinical characteristics before and after propensity score matching (PSM) of patients from SEER database.
**Table S3:** Clinical characteristics before and after propensity score matching (PSM) of patients from SEER database.
**Table S4:** Clinical characteristics in raw data of cT3cN0cM0 Patients in the SEER Database.
**Table S5:** Clinical characteristics before and after propensity score matching (PSM) of cT3cN0cM0 patients from SEER database.
**Table S6:** Influence of Different Neck Dissection Strategies on PFS under Cox Regression Models in the SYSU Database.

## Data Availability

The first part of this article is based on data from the SEER database, which can be accessed by applying for an account on the SEER website and using the SEER*Stat software (https://seer.cancer.gov.seerstat/). Data from our hospital used for the second part of this article are available on request by emailing huangbx5@mail2.sysu.edu.cn.
