# Supplementary figures and images for: Bilateral Neck Dissection Effectively Improves Prognosis of Patients With T3N0M0 Glottic Carcinoma
Source: Cancer Med. 2026 Feb 1;15(2):e71593. doi: 10.1002/cam4.71593 (PMC12861563; doi:10.1002/cam4.71593)

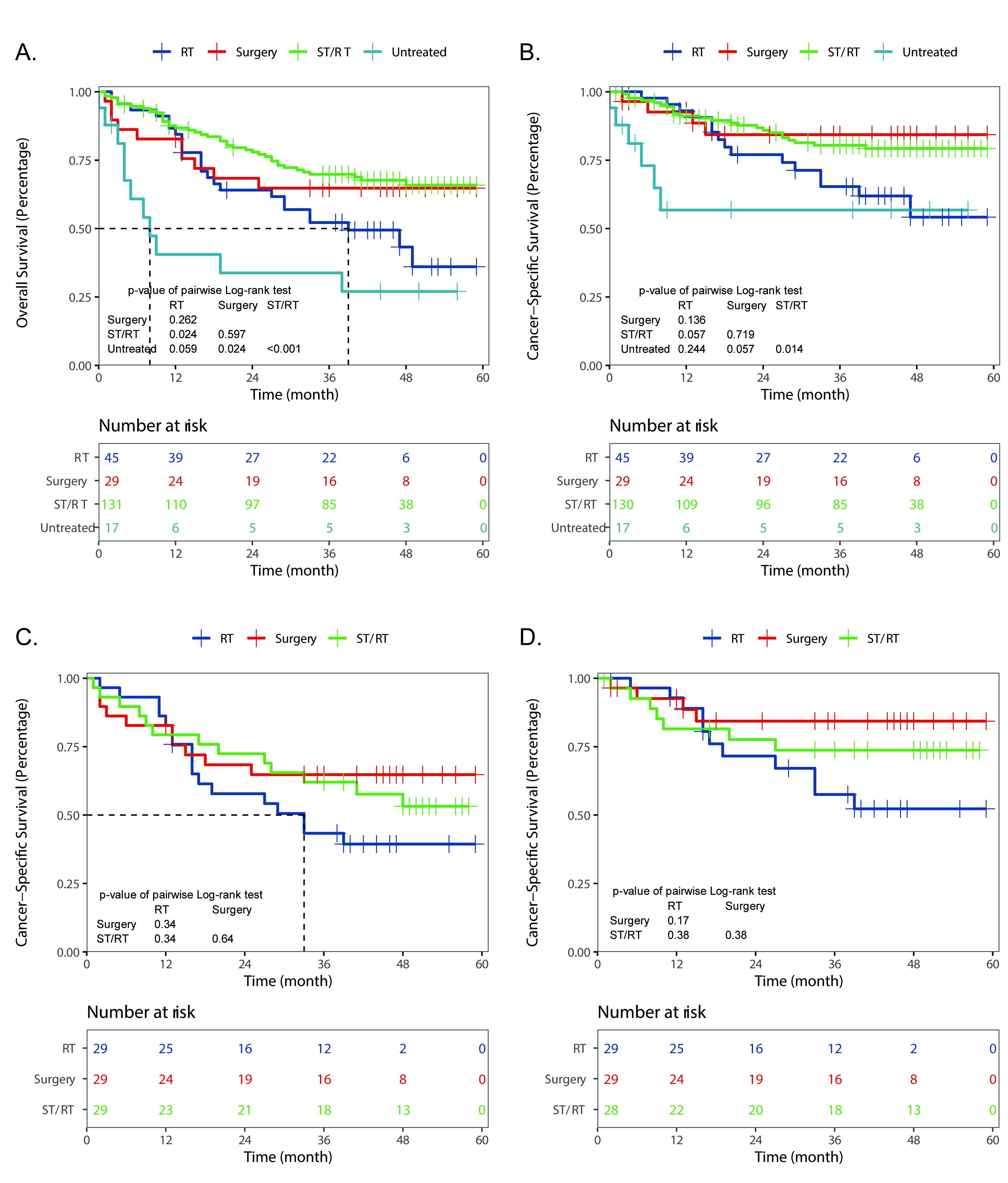

Supplement: Supplementary file 1 — Figure S1: Kaplan–Meier survival curves for patient with cT3cN0cM0 in OS (A, C) and CSS (B, D) among different treatment group in raw data (A, B), data after PSM (C, D). [file CAM4-15-e71593-s003.tif]

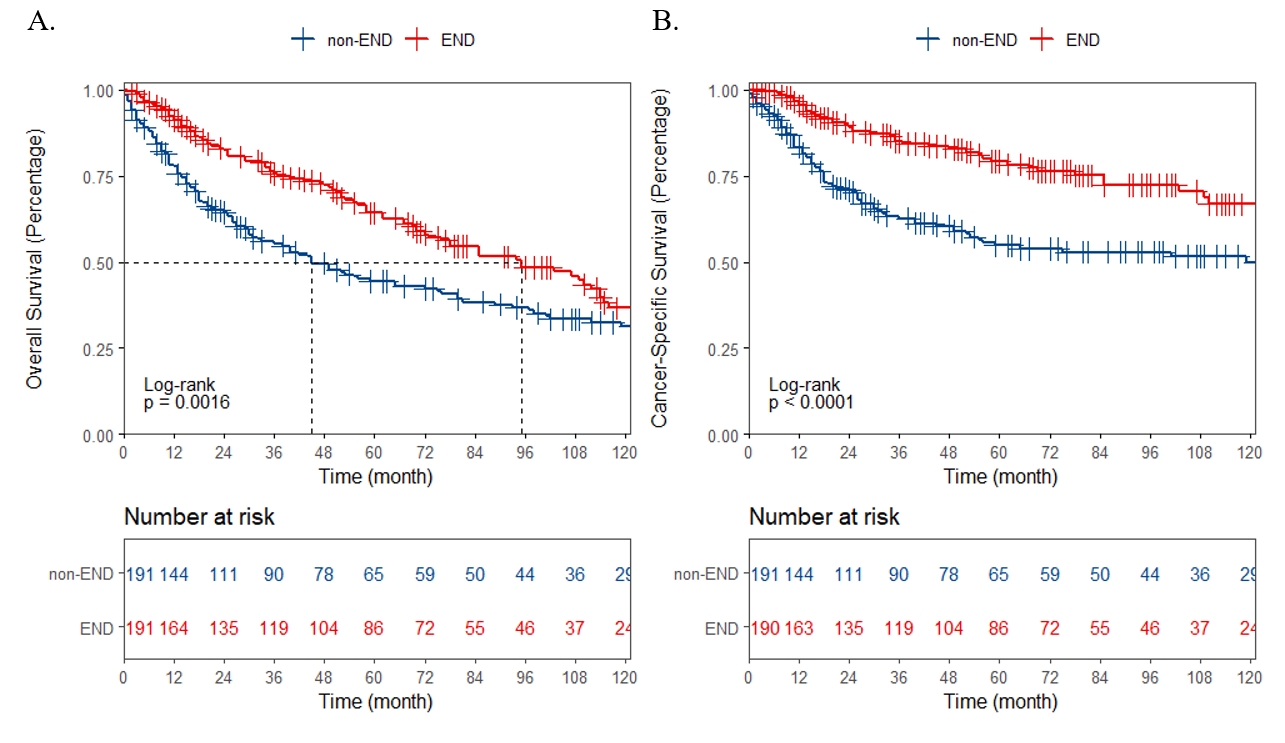

Supplement: Supplementary file 2 — Figure S2: Kaplan–Meier survival curves for patient with and without END in OS (A) and CSS (B), data after PSM. [file CAM4-15-e71593-s002.jpg]
