# Supplementary material for: Bilateral Neck Dissection Effectively Improves Prognosis of Patients With T3N0M0 Glottic Carcinoma
Source: Cancer Med. 2026 Feb 1;15(2):e71593. doi: 10.1002/cam4.71593 (PMC12861563; doi:10.1002/cam4.71593)
Supplement: Supplementary file 3 — Table S1: Clinical characteristics in raw data from SEER database. Table S2: Clinical characteristics before and after propensity score matching (PSM) of patients from SEER database. Table S3: Clinical characteristics before and after propensity score matching (PSM) of patients from SEER database. Table S4: Clinical characteristics in raw data of cT3cN0cM0 Patients in the SEER Database. Table S5: Clinical characteristics before and after propensity score matching (PSM) of cT3cN0cM0 patients from SEER database. Table S6: Influence of Different Neck Dissection Strategies on PFS under Cox Regression Models in the SYSU Database. [file CAM4-15-e71593-s001.docx]

**Supplementary Table**

Supplementary Table 1. Clinical characteristics in raw data from SEER database.

| **Variables** | **Total**  **(n = 2027)** | **Method** | | | | | **Statistic** | ***P*** |
| --- | --- | --- | --- | --- | --- | --- | --- | --- |
|  |  | **Untreated**  **(n = 158)** | **Radiation Therapy**  **(n = 336)** | **Surgery**  **(n = 614)** | **Systemic Therapy/RT**  **(n = 919)** |  |  |  |
| **Year of diagnosis, Mean ± SD** | 2012.28 ± 4.81 | 2011.73 ± 4.59 | 2012.13 ± 4.69 | 2011.68 ± 4.83 | 2012.82 ± 4.82 |  | F=8.02 | **<.001** |
| **Sex, n (%)** |  |  |  |  |  |  | χ²=2.56 | 0.465 |
| Male | 1773 (87.47) | 143 (90.51) | 297 (88.39) | 529 (86.16) | 804 (87.49) |  |  |  |
| Female | 254 (12.53) | 15 (9.49) | 39 (11.61) | 85 (13.84) | 115 (12.51) |  |  |  |
| **Age, n (%)** |  |  |  |  |  |  | χ²=74.23 | **<.001** |
| <65 | 892 (44.01) | 41 (25.95) | 96 (28.57) | 284 (46.25) | 471 (51.25) |  |  |  |
| ≥65 | 1135 (55.99) | 117 (74.05) | 240 (71.43) | 330 (53.75) | 448 (48.75) |  |  |  |
| **Race, n (%)** |  |  |  |  |  |  | χ²=7.06 | 0.316 |
| White | 1547 (76.32) | 123 (77.85) | 263 (78.27) | 479 (78.01) | 682 (74.21) |  |  |  |
| Black | 366 (18.06) | 25 (15.82) | 55 (16.37) | 110 (17.92) | 176 (19.15) |  |  |  |
| Others | 114 (5.62) | 10 (6.33) | 18 (5.36) | 25 (4.07) | 61 (6.64) |  |  |  |
| **Laterality, n (%)** |  |  |  |  |  |  | χ²=7.59 | 0.576 |
| Left | 239 (11.79) | 19 (12.03) | 39 (11.61) | 63 (10.26) | 118 (12.84) |  |  |  |
| Right | 244 (12.04) | 20 (12.66) | 43 (12.80) | 67 (10.91) | 114 (12.40) |  |  |  |
| Bilateral | 18 (0.89) | 1 (0.63) | 2 (0.60) | 3 (0.49) | 12 (1.31) |  |  |  |
| Not a paired site | 1526 (75.28) | 118 (74.68) | 252 (75.00) | 481 (78.34) | 675 (73.45) |  |  |  |
| **Grade, n (%)** |  |  |  |  |  |  | χ²=39.19 | **<.001** |
| Well differentiated | 259 (12.78) | 15 (9.49) | 44 (13.10) | 91 (14.82) | 109 (11.86) |  |  |  |
| Moderately differentiated | 905 (44.65) | 68 (43.04) | 150 (44.64) | 297 (48.37) | 390 (42.44) |  |  |  |
| Poorly differentiated | 225 (11.10) | 19 (12.03) | 31 (9.23) | 85 (13.84) | 90 (9.79) |  |  |  |
| Undifferentiated | 7 (0.35) | 2 (1.27) | 1 (0.30) | 2 (0.33) | 2 (0.22) |  |  |  |
| Unknown | 631 (31.13) | 54 (34.18) | 110 (32.74) | 139 (22.64) | 328 (35.69) |  |  |  |

χ²: Chi-square test, F: ANOVA, SD: standard deviation

Supplementary Table 2. Clinical characteristics before and after propensity score matching (PSM) of patients from SEER database.

| **Variables** | **Before PSM** | | | | | |  | **After PSM** | | | | | |
| --- | --- | --- | --- | --- | --- | --- | --- | --- | --- | --- | --- | --- | --- |
|  | **Total**  **(n = 1524)** | **Method** | | | **Statistic** | ***P*** |  | **Total**  **(n = 696)** | **Method** | | | **Statistic** | ***P*** |
|  |  | **non-END**  **(n = 373)** | **END**  **(n = 232)** | **Systemic Therapy/RT**  **(n = 919)** |  |  |  |  | **non-END**  **(n = 232)** | **END**  **(n = 232)** | **Systemic Therapy/RT**  **(n = 232)** |  |  |
| **Sex, n (%)** |  |  |  |  | χ²=0.69 | 0.707 |  |  |  |  |  | χ²=0.18 | 0.914 |
| Male | 1325 (86.94) | 320 (85.79) | 201 (86.64) | 804 (87.49) |  |  |  | 607 (87.21) | 204 (87.93) | 201 (86.64) | 202 (87.07) |  |  |
| Female | 199 (13.06) | 53 (14.21) | 31 (13.36) | 115 (12.51) |  |  |  | 89 (12.79) | 28 (12.07) | 31 (13.36) | 30 (12.93) |  |  |
| **Age, n (%)** |  |  |  |  | χ²=14.83 | **<.001** |  |  |  |  |  | χ²=0.00 | 1.000 |
| ＜65 | 752 (49.34) | 153 (41.02) | 128 (55.17) | 471 (51.25) |  |  |  | 384 (55.17) | 128 (55.17) | 128 (55.17) | 128 (55.17) |  |  |
| ≥65 | 772 (50.66) | 220 (58.98) | 104 (44.83) | 448 (48.75) |  |  |  | 312 (44.83) | 104 (44.83) | 104 (44.83) | 104 (44.83) |  |  |
| **Race, n (%)** |  |  |  |  | χ²=5.44 | 0.245 |  |  |  |  |  | χ²=0.86 | 0.930 |
| White | 1154 (75.72) | 294 (78.82) | 178 (76.72) | 682 (74.21) |  |  |  | 541 (77.73) | 181 (78.02) | 178 (76.72) | 182 (78.45) |  |  |
| Black | 284 (18.64) | 64 (17.16) | 44 (18.97) | 176 (19.15) |  |  |  | 126 (18.10) | 43 (18.53) | 44 (18.97) | 39 (16.81) |  |  |
| Others | 86 (5.64) | 15 (4.02) | 10 (4.31) | 61 (6.64) |  |  |  | 29 (4.17) | 8 (3.45) | 10 (4.31) | 11 (4.74) |  |  |
| **Laterality, n (%)** |  |  |  |  | χ²=16.08 | **0.013** |  |  |  |  |  | - | 0.974 |
| Left | 180 (11.81) | 38 (10.19) | 24 (10.34) | 118 (12.84) |  |  |  | 72 (10.34) | 23 (9.91) | 24 (10.34) | 25 (10.78) |  |  |
| Right | 178 (11.68) | 51 (13.67) | 13 (5.60) | 114 (12.40) |  |  |  | 37 (5.32) | 14 (6.03) | 13 (5.60) | 10 (4.31) |  |  |
| Bilateral | 15 (0.98) | 2 (0.54) | 1 (0.43) | 12 (1.31) |  |  |  | 4 (0.57) | 1 (0.43) | 1 (0.43) | 2 (0.86) |  |  |
| Not a paired site | 1151 (75.52) | 282 (75.60) | 194 (83.62) | 675 (73.45) |  |  |  | 583 (83.76) | 194 (83.62) | 194 (83.62) | 195 (84.05) |  |  |
| **Grade, n (%)** |  |  |  |  | χ²=32.54 | **<.001** |  |  |  |  |  | χ²=2.27 | 0.972 |
| Well differentiated | 197 (12.93) | 58 (15.55) | 30 (12.93) | 109 (11.86) |  |  |  | 89 (12.79) | 31 (13.36) | 30 (12.93) | 28 (12.07) |  |  |
| Moderately differentiated | 684 (44.88) | 178 (47.72) | 116 (50.00) | 390 (42.44) |  |  |  | 344 (49.43) | 113 (48.71) | 116 (50.00) | 115 (49.57) |  |  |
| Poorly Undifferentiated | 173 (11.35) | 47 (12.60) | 36 (15.52) | 90 (9.79) |  |  |  | 109 (15.66) | 37 (15.95) | 36 (15.52) | 36 (15.52) |  |  |
| Undifferentiated | 4 (0.26) | 1 (0.27) | 1 (0.43) | 2 (0.22) |  |  |  | 3 (0.43) | 0 (0.00) | 1 (0.43) | 2 (0.86) |  |  |
| Unknown | 466 (30.58) | 89 (23.86) | 49 (21.12) | 328 (35.69) |  |  |  | 151 (21.70) | 51 (21.98) | 49 (21.12) | 51 (21.98) |  |  |

χ²: Chi-square test, -: Fisher exact

Supplementary Table 3. Clinical characteristics before and after propensity score matching (PSM) of patients from SEER database.

| **Variables** | **Before PSM** | | | | |  | **After PSM** | | | | |
| --- | --- | --- | --- | --- | --- | --- | --- | --- | --- | --- | --- |
|  | **Total**  **(n = 605)** | **Elective node dissection** | | **Statistic** | ***P*** |  | **Total**  **(n = 382)** | **Elective node dissection** | | **Statistic** | ***P*** |
|  |  | **non-END**  **(n = 373)** | **END**  **(n =232)** |  |  |  |  | **non-END**  **(n = 191)** | **END**  **(n = 191)** |  |  |
| **Sex, n (%)** |  |  |  | χ²=0.086 | 0.770 |  |  |  |  | χ²=0.293 | 0.588 |
| Male | 521 (86.12) | 320 (85.79) | 201 (86.64) |  |  |  | 316 (82.72) | 156 (81.68) | 160 (83.77) |  |  |
| Female | 84 (13.88) | 53 (14.21) | 31 (13.36) |  |  |  | 66 (17.28) | 35 (18.32) | 31 (16.23) |  |  |
| **Age, n (%)** |  |  |  | χ²=11.520 | **<.001** |  |  |  |  | χ²=0.000 | 1.000 |
| ＜65 | 281 (46.45) | 153 (41.02) | 128 (55.17) |  |  |  | 174 (45.55) | 87 (45.55) | 87 (45.55) |  |  |
| ≥65 | 324 (53.55) | 220 (58.98) | 104 (44.83) |  |  |  | 208 (54.45) | 104 (54.45) | 104 (54.45) |  |  |
| **Race, n (%)** |  |  |  | χ²=0.371 | 0.831 |  |  |  |  | χ²=2.569 | 0.277 |
| White | 472 (78.02) | 294 (78.82) | 178 (76.72) |  |  |  | 297 (77.75) | 155 (81.15) | 142 (74.35) |  |  |
| Black | 108 (17.85) | 64 (17.16) | 44 (18.97) |  |  |  | 68 (17.8) | 29 (15.18) | 39 (20.42) |  |  |
| Others | 25 (4.13) | 15 (4.02) | 10 (4.31) |  |  |  | 17 (4.45) | 7 (3.66) | 10 (5.24) |  |  |
| **Laterality , n (%)** |  |  |  | - | **0.009** |  |  |  |  | - | 0.595 |
| Left | 62 (10.25) | 38 (10.19) | 24 (10.34) |  |  |  | 38 (9.95) | 15 (7.85) | 23 (12.04) |  |  |
| Right | 64 (10.58) | 51 (13.67) | 13 (5.60) |  |  |  | 26 (6.81) | 13 (6.81) | 13 (6.81) |  |  |
| Bilateral | 3 (0.5) | 2 (0.54) | 1 (0.43) |  |  |  | 2 (0.52) | 1 (0.52) | 1 (0.52) |  |  |
| **Grade, n (%)** |  |  |  | χ²=2.300 | 0.681 |  |  |  |  | χ²=1.373 | 0.849 |
| Well differentiated | 88 (14.55) | 58 (15.55) | 30 (12.93) |  |  |  | 51 (13.35) | 24 (12.57) | 27 (14.14) |  |  |
| Moderately differentiated | 294 (48.6) | 178 (47.72) | 116 (50.00) |  |  |  | 189 (49.48) | 94 (49.21) | 95 (49.74) |  |  |
| Poorly differentiated | 83 (13.72) | 47 (12.60) | 36 (15.52) |  |  |  | 65 (17.02) | 34 (17.80) | 31 (16.23) |  |  |
| Undifferentiated | 2 (0.33) | 1 (0.27) | 1 (0.43) |  |  |  | 1 (0.26) | 0 (0.00) | 1 (0.52) |  |  |
| Unknown | 138 (22.81) | 89 (23.86) | 49 (21.12) |  |  |  | 76 (19.9) | 39 (20.42) | 37 (19.37) |  |  |
| **Radiation Therapy, n (%)** |  |  |  | χ²=56.012 | **<.001** |  |  |  |  | χ²=0.011 | 0.918 |
| Yes | 393 (64.96) | 285 (76.41) | 108 (46.55) |  |  |  | 217 (56.81) | 109 (57.07) | 108 (56.54) |  |  |
| No | 212 (35.04) | 88 (23.59) | 124 (53.45) |  |  |  | 165 (43.19) | 82 (42.93) | 83 (43.46) |  |  |

χ²: Chi-square test, -: Fisher exact

Supplementary Table 4. Clinical characteristics in raw data of cT3cN0cM0 Patients in the SEER Database

| **Variables** | **Total**  **(n = 222)** | **Method** | | | | **Statistic** | **P** |
| --- | --- | --- | --- | --- | --- | --- | --- |
|  |  | **Untreated**  **(n = 17)** | **Radiation Therapy**  **(n = 45)** | **Surgery**  **(n = 29)** | **Systemic Therapy/RT**  **(n = 131)** |  |  |
| **Age, n(%)** |  |  |  |  |  | χ²=14.42 | **0.002** |
| ＜65 | 92 (41.44) | 9 (52.94) | 8 (17.78) | 11 (37.93) | 64 (48.85) |  |  |
| ≥65 | 130 (58.56) | 8 (47.06) | 37 (82.22) | 18 (62.07) | 67 (51.15) |  |  |
| **Sex, n(%)** |  |  |  |  |  | - | 0.135 |
| Male | 185 (83.33) | 17 (100.00) | 36 (80.00) | 22 (75.86) | 110 (83.97) |  |  |
| Female | 37 (16.67) | 0 (0.00) | 9 (20.00) | 7 (24.14) | 21 (16.03) |  |  |
| **Race, n(%)** |  |  |  |  |  | - | 0.343 |
| White | 158 (71.17) | 13 (76.47) | 38 (84.44) | 20 (68.97) | 87 (66.41) |  |  |
| Black | 46 (20.72) | 2 (11.76) | 5 (11.11) | 7 (24.14) | 32 (24.43) |  |  |
| Others | 18 (8.11) | 2 (11.76) | 2 (4.44) | 2 (6.90) | 12 (9.16) |  |  |
| **Laterality, n(%)** |  |  |  |  |  | - | 0.504 |
| Left | 50 (22.52) | 5 (29.41) | 11 (24.44) | 6 (20.69) | 28 (21.37) |  |  |
| Right | 36 (16.22) | 1 (5.88) | 5 (11.11) | 9 (31.03) | 21 (16.03) |  |  |
| Bilateral | 1 (0.45) | 0 (0.00) | 0 (0.00) | 0 (0.00) | 1 (0.76) |  |  |
| Not a paired site | 135 (60.81) | 11 (64.71) | 29 (64.44) | 14 (48.28) | 81 (61.83) |  |  |
| **Grade, n(%)** |  |  |  |  |  | - | 0.142 |
| Well differentiated | 33 (14.86) | 3 (17.65) | 7 (15.56) | 7 (24.14) | 16 (12.21) |  |  |
| Moderately differentiated | 121 (54.50) | 11 (64.71) | 23 (51.11) | 13 (44.83) | 74 (56.49) |  |  |
| Poorly differentiated | 24 (10.81) | 0 (0.00) | 5 (11.11) | 7 (24.14) | 12 (9.16) |  |  |
| Undifferentiated | 44 (19.82) | 3 (17.65) | 10 (22.22) | 2 (6.90) | 29 (22.14) |  |  |
| χ²: Chi-square test, -: Fisher exact | | | | | | | |

Supplementary Table 5. Clinical characteristics before and after propensity score matching (PSM) of cT3cN0cM0 patients from SEER database.

| **Variables** | **Before PSM** | | | | | |  |  | **After PSM** | | |  |  |
| --- | --- | --- | --- | --- | --- | --- | --- | --- | --- | --- | --- | --- | --- |
|  | **Total**  **(n = 205)** | **Method** | | | **Statistic** | ***P*** |  | **Total**  **(n = 87)** | **Method** | | | **Statistic** | ***P*** |
|  |  | **Radiation Therapy**  **(n = 45)** | **Surgery**  **(n = 29)** | **Systemic Therapy/RT**  **(n = 131)** |  |  |  |  | **Radiation Therapy**  **(n = 29)** | **Surgery**  **(n = 29)** | **Systemic Therapy/RT**  **(n = 29)** |  |  |
| **Age, n(%)** |  |  |  |  | χ²=13.52 | **0.001** |  |  |  |  |  | χ²=0.97 | 0.617 |
| ＜65 | 83 (40.49) | 8 (17.78) | 11 (37.931) | 64 (48.86) |  |  |  | 27 (31.03) | 8 (27.59) | 11 (37.93) | 8 (27.59) |  |  |
| ≥65 | 122 (59.51) | 37 (82.22) | 18 (62.069) | 67 (51.15) |  |  |  | 60 (68.97) | 21 (72.41) | 18 (62.07) | 21 (72.41) |  |  |
| **Sex, n(%)** |  |  |  |  | χ²=1.20 | 0.548 |  |  |  |  |  | χ²=0.13 | 0.937 |
| Male | 168 (81.95) | 36 (80.00) | 22 (75.862) | 110 (83.97) |  |  |  | 67 (77.01) | 23 (79.31) | 22 (75.86) | 22 (75.86) |  |  |
| Female | 37 (18.05) | 9 (20.00) | 7 (24.138) | 21 (16.03) |  |  |  | 20 (22.99) | 6 (20.69) | 7 (24.14) | 7 (24.14) |  |  |
| **Race, n(%)** |  |  |  |  | - | 0.242 |  |  |  |  |  | - | 0.941 |
| White | 145 (70.73) | 38 (84.44) | 20 (68.966) | 87 (66.412) |  |  |  | 64 (73.56) | 22 (75.86) | 20 (68.97) | 22 (75.86) |  |  |
| Black | 44 (21.46) | 5 (11.11) | 7 (24.15) | 32 (24.43) |  |  |  | 18 (20.69) | 5 (17.24) | 7 (24.14) | 6 (20.69) |  |  |
| Others | 16 (7.80) | 2 (4.44) | 2 (6.90) | 12 (9.16) |  |  |  | 5 (5.75) | 2 (6.90) | 2 (6.90) | 1 (3.45) |  |  |
| **Laterality, n(%)** |  |  |  |  | - | 0.420 |  |  |  |  |  | χ²=3.01 | 0.556 |
| Left | 45 (21.95) | 11 (24.44) | 6 (20.69) | 28 (21.37) |  |  |  | 23 (26.44) | 9 (31.03) | 6 (20.69) | 8 (27.59) |  |  |
| Right | 35 (17.07) | 5 (11.11) | 9 (31.03) | 21 (16.03) |  |  |  | 24 (27.59) | 5 (17.24) | 9 (31.03) | 10 (34.48) |  |  |
| Bilateral | 1 (0.49) | 0 (0.00) | 0 (0.00) | 1 (0.76) |  |  |  | 0 (0.00) | 0 (0.00) | 0 (0.00) | 0 (0.00) |  |  |
| Not a paired site | 124 (60.49) | 29 (64.44) | 14 (48.28) | 81 (61.83) |  |  |  | 40 (45.98) | 15 (51.72) | 14 (48.28) | 11 (37.93) |  |  |
| **Grade, n(%)** |  |  |  |  | χ²=10.48 | 0.106 |  |  |  |  |  | - | 0.815 |
| Well differentiated | 30 (14.63) | 7 (15.56) | 7 (24.14) | 16 (12.21) |  |  |  | 23 (26.44) | 7 (24.14) | 7 (24.14) | 9 (31.03) |  |  |
| Moderately differentiated | 110 (53.66) | 23 (51.11) | 13 (44.83) | 74 (56.49) |  |  |  | 34 (39.08) | 12 (41.38) | 13 (44.83) | 9 (31.03) |  |  |
| Poorly Undifferentiated | 24 (11.71) | 5 (11.11) | 7 (24.14) | 12 (9.16) |  |  |  | 18 (20.69) | 5 (17.24) | 7 (24.14) | 6 (20.69) |  |  |
| Undifferentiated | 41 (20.00) | 10 (22.22) | 2 (6.90) | 29 (22.14) |  |  |  | 12 (13.79) | 5 (17.24) | 2 (6.90) | 5 (17.24) |  |  |
| χ²: Chi-square test, -: Fisher exact | | | | | | | | | | | | | |

Supplementary Table 6. Influence of Different Neck Dissection Strategies on PFS under Cox Regression Models in the SYSU Database.

| **Variables** | **Model 1** | |  | **Model 2** | |
| --- | --- | --- | --- | --- | --- |
|  | **HR (95%CI)** | ***P*** |  | **HR (95%CI)** | ***P*** |
| **Method** |  |  |  |  |  |
| Bilateral | 1.00 (Reference) |  |  | 1.00 (Reference) |  |
| Unilateral | 3.05 (1.11 ~ 8.40) | **0.031** |  | 3.79 (1.16 ~ 12.42) | **0.028** |
| HR: Hazard Ratio, CI: Confidence Interval  Model 1: Crude  Model 2: Adjust: Sex, age, Surgical, Grade, Laterality, ECOG Performance Status, Adjuvant radiotherapy | | | | | |
